# VAV2-associated ncRNA network in the focal adhesion pathway is dysregulated in laryngeal squamous cell carcinoma

**DOI:** 10.1007/s12672-026-05181-z

**Published:** 2026-05-27

**Authors:** Payam Mohammadi, Najmeh Parvaz, Fariba MehdiKhani, Mohammad Elahimanesh, Masoomeh Bakhshandeh, Keyvan Aghazadeh, Mohsen Aryan Tabar, Mohammad Shabani, Mohammad Najafi

**Affiliations:** 1 Department of Biochemistry, School of Medicine, Iran University of Medical Sciences, Tehran, Iran; 2 Clinical Biochemistry Department, Faculty of Medical Sciences, Tehran University of Medical Sciences, Tehran, Iran; 3 Otorhinolaryngology Research Center, Amir Alam Hospital, Tehran University of Medical Sciences, Tehran, Iran; 4 Department of Biochemistry, Faculty of Medicine, Baqiyatallah University of Medical Sciences, Tehran, Iran

**Keywords:** Laryngeal squamous cell carcinoma, VAV2, Network, LINC00665, CCDC144NL-AS1, MiR-449b-5p, MiR-495-3p, Focal adhesion, TCGA, GEO

## Abstract

**Background:**

The focal adhesion is a key pathway for cellular proliferation and migration. This study aimed to elucidate the function of VAV2, a key guanine nucleotide exchange factor in the focal adhesion pathway, and to investigate its post-transcriptional relationships through the predicted VAV2/miRNA/lncRNA network in Laryngeal Squamous Cell Carcinoma (LSCC).

**Methods:**

RNA-seq data analysis from The Cancer Genome Atlas (TCGA) and Gene Expression Omnibus (GEO) datasets was performed using R software. Differential expression profiles of mRNAs, miRNAs, and lncRNAs were integrated with multi-database miRNA-target predictions to construct a high-confidence VAV2-associated ncRNA network. The RT-qPCR and Western blotting techniques were used to validate the gene analysis in 63 paired LSCC and adjacent normal tissues.

**Results:**

Focal adhesion was among the significantly enriched pathways (47 genes, *P* = 2.23 × 10⁻⁴). VAV2 exhibited consistent upregulation at both mRNA and protein levels in tumor tissues (*P* < 0.0001). Tumor samples exhibited the downregulation of miR-449b-5p and miR-495-3p, along with pronounced significant overexpression of LINC00665 and CCDC144NL-AS1 (*P* < 0.01). Significant positive correlations were identified between VAV2 and lncRNAs (*r* = 0.76 and *r* = 0.79, *P* < 0.0001), alongside comparable negative correlations with miRNAs.

**Conclusion:**

Our results proposed that the predicted VAV2-associated ncRNA network might be involved in the focal adhesion pathway. These data suggested novel insights into the underlying mechanisms and hold promise as biomarkers and therapeutic targets.

**Supplementary Information:**

The online version contains supplementary material available at 10.1007/s12672-026-05181-z.

## Introduction

A considerable proportion of head and neck squamous cell carcinoma (HNSCC) cases arise as laryngeal squamous cell carcinoma (LSCC), a major malignancy of the upper aerodigestive tract [1]. LSCC exhibits a striking gender disparity, occurring nearly seven times more often in males than females, underscoring its significance as a global public health concern [2]. The pathogenesis is multifaceted, encompassing interactions among genetic predisposition, epigenetic modifications, and lifestyle factors such as tobacco and alcohol consumption [3].

Accordingly, a detailed understanding of the biological mechanisms driving laryngeal carcinogenesis is critical for the development of more effective diagnostic approaches, preventive strategies, and therapeutic interventions that may improve patient survival [4]. Accurate diagnosis and clinical staging provide the foundation for treatment planning and prognostication in LSCC [5]. Early-stage LSCC is typically managed with surgery or radiotherapy, whereas advanced stages often require multimodal strategies aimed at balancing oncologic control with laryngeal preservation [6]. Molecular and cellular diagnostics are becoming more widely used as adjunctive approaches, enabling earlier intervention and more reliable detection of persistent or recurrent disease, especially after radiotherapy, where tissue alterations caused by radiation may obscure conventional evaluation [7]. Understanding the cellular and genetic alterations underlying LSCC progression may further enable the development of targeted therapies. In this context, high-throughput techniques such as RNA sequencing have enabled comprehensive molecular profiling, identified biomarkers for early detection, and tailored treatment approaches [8, 9]. Through computational analysis of large molecular datasets, bioinformatics plays a key role in identifying tumor-specific markers and advancing early diagnosis and molecular cancer research [10–13]. Public repositories such as TCGA and GEO provide extensive transcriptomic resources for such investigations [14].

The focal adhesion signaling pathway is involved in cellular invasion, proliferation, and migration; however, the specific roles of many of its genes in cancer biology remain unclear [15]. Among these genes, VAV2 and PAK2 [16] are reported to play critical roles in oncogenic signaling through enhanced focal adhesion turnover. Overexpression of VAV2 has been consistently linked to tumor invasion, metastasis, and poor prognosis in breast [17, 18], gastric [19], lung [20], adrenocortical [21, 22], and head-and-neck squamous cell carcinoma [23, 24]. Moreover, regulation of pathways via genes is influenced by transcription factors, epigenetic mechanisms, and non-coding RNAs [25–27]. Transcriptomic alterations in cancer extend well beyond protein-coding genes, and recent research highlights the central role of non-coding RNAs in tumor initiation and progression [28]. Long Non-coding RNAs (lncRNAs) and microRNAs (miRNAs) are two principal types that substantially influence oncogenesis and progression of LSCC [29]. According to the competing endogenous RNA (ceRNA) hypothesis, lncRNAs may function as molecular sponges that bind and sequester miRNAs, thereby relieving their repression of target mRNAs and modulating cancer-related gene expression programs [30]. Numerous experimental investigations have validated the biological relevance of lncRNA/miRNA/mRNA networks and highlighted their promise as diagnostic, prognostic, and therapeutic markers in LSCC [31–35].

In this study, we performed an integrated bioinformatics analysis of TCGA and GEO transcriptomic datasets to characterize the expression pattern, biological relevance, and survival probability of VAV2 in LSCC. To better understand the regulation of VAV2 in the focal adhesion pathway, we applied systems biology analyses to construct a VAV2/ncRNA network and measured the expression levels of LINC00665, miR-449b-5p, CCDC144NL-AS1, miR-495-3p, and VAV2.

## Materials and methods

### Differentially expressed genes (DEGs)

RNA‑seq data were obtained from TCGA, including 100 laryngeal tumor samples and 11 matched adjacent normal tissues. Additional datasets were identified in GEO by searching the terms “laryngeal squamous cell carcinoma (LSCC)” and “paired adjacent normal tissues”, restricted to studies published and updated within the past five years (GSE130605, tumor–normal pairs (*n* = 50)). To identify differentially expressed ncRNAs, expression data were collected from the GEO datasets GSE132222, GSE133632, GSE137308, GSE142083, GSE127165, and GSE216664 using the keywords miRNA, lncRNA, non‑coding RNA, and laryngeal squamous cell carcinoma (LSCC). The differentially expressed genes (DEmiRs (|log₂FC| ≤ −1), DEGs, and DElncRNAs (log₂FC ≥ 1), adjusted *p* < 0.05) were identified using DESeq2 (v1.49.3) in R v4.3.3 after a meta-analysis performed across the datasets.

### Pathway enrichment

To detect dysregulated signaling cascades associated with LSCC tumor progression, the intersection genes were consistently identified as upregulated DEGs (log₂FC ≥ 1, *p* < 0.05) to enrich pathways in the WikiPathway database in DIVID Bioinformatics Resource. Focal adhesion was among the significantly enriched pathways, and VAV2 emerged as a centrally positioned, upregulated gene. Additional enrichment analyses were performed using tools of clusterProfiler (v4.17.0), ggplot2 (v3.5.2), and ComplexHeatmap (v2.24.1) packages in R for annotation, visualization, and integrated discovery, allowing systematic identification of enriched functional categories and biological processes.

### Gene/miRNA/lncRNA network

The curated experimental (miRTarBase, StarBase, and DIANA-TarBase) and predictive (TargetScan, miRWalk, miRDB, and miRmap) databases were used to reveal miRNA– lncRNA and VAV2 relationships. The data were systematically supported by additional resources containing DElncRNAs and DEmiRs from the GEO dataset. A weighted scoring approach was used to evaluate interaction confidence. Curated experimental database reports were assigned a score of 1, whereas predictive database reports were assigned a score of 0.5. The total score for each interaction was computed by integrating the normalized mean fold‑change values with the corresponding experimental and predictive database report weights (Supplements 1, 2, and 3). A Gene (VAV2)/miRNA/lncRNA network was constructed to characterize potential regulatory interactions using Cytoscape (v3.10.3). The network edges and lncRNA nodes were visualized based on the total score.

### Tissue samples

A total of 63 paired LSCC and adjacent non-malignant tissue samples were obtained through candidates for surgery with pathologically confirmed diagnoses from the Tumor Bank of the Otorhinolaryngology Research Center, Amir Alam Hospital (Tehran, Iran). Tissue specimens were transferred to the molecular lab in liquid nitrogen and stored at − 80 °C. The study was approved by the Ethics Committee of Iran University of Medical Sciences (IR.IUMS.FMD.REC.1402.460).

### RNA extraction and gene expression

Total RNA was isolated from all tissue specimens using the Extraction and Purification Kit (Sinaclon, Iran, Cat. No. EX6051), following the manufacturer’s instructions. The purified RNA was subsequently reverse-transcribed into complementary DNA (cDNA) using the Yektatajhiz cDNA Synthesis Kit (Yektatajhiz, Iran, Cat. No. YT4500). For mature miRNA, cDNA synthesis was performed with a miRNA-specific stem-loop primer. Relative mRNA, miRNA, and lncRNA expression levels were calculated using the 2^−ΔΔCt^ method. RT-qPCR technique was performed on the StepOne Real-Time PCR Detection System (Applied Biosystems, USA) using the Yektatajhiz SYBR Green qPCR Master Mix (Yektatajhiz, Tehran, Iran, Cat. No. YT2551). GAPDH served as the reference gene for mRNA and lncRNA, whereas U6 was used as the reference gene for miRNAs. Primer design was conducted using NCBI Primer-BLAST (https://www.ncbi.nlm.nih.gov/tools/ primer- blast/)), and was verified through OligoAnalyzer software (version 1.0.2 [36]. All primer sequences are provided in Table 1.

**Table 1:** Gene and ncRNA primers

Gene/ncRNA/Loop	Primer sequences (5’−3’)
VAV2	Forward: GGCGAGACCAACGGACGGA Reverse: ATTCTCAACACCCTCCCTATCCCTG
miR-449b-5p	Forward: CGTGGTTGGAGGCAGTGTATTGTT Reverse: CGTTGGCTCTGGTGCTGGGT Stem Loop: CGTTGGCTCTGGTGCTGGGTCCGAGGTATTCGCACCAGAGCCAACGGCCAGCT
miR-495-3p	Forward: GGCGAGGGAAACAAACATGGTGC Reverse: CGTTGGCTCTGGTGCTGGGT Stem Loop: CGTTGGCTCTGGTGCTGGGTCCGAGGTATTCGCACCAGAGCCAACGAAGAAGT
CCDC144NL-AS1	Forward: GACCAATCTTCCTCTTCCTCCTTCT Reverse: GTCTACGCTGATGGCTGCTG
LINC00665	Forward: CGCTGATGTAGTTTCCTGACCT Reverse: ACTCAGAGGTGGAATTTTTGTGGG
GAPDH	Forward: CATGAGAAGTATGACAACAGCC Reverse: AGTCCTTCCACGATACCAAAGT
U6	Forward: TTGGAACGATACAGAGAAGATTAGC Reverse: TATGGAACGCTTCACGAATTTGC

### Protein expression

Total protein samples were extracted from LSCC and adjacent normal tissue samples using ice-cold RIPA lysis buffer supplemented with protease inhibitors. Lysates were centrifuged at 14,000 × g for 20 min at 4 °C to remove cellular debris. Protein values were measured using the Bradford Protein Assay Kit (DNAbioTech, Iran; Cat. No. DB0017) according to the manufacturer’s protocol. 20 µg protein was mixed with 2× Laemmli sample buffer, boiled for 5 min, and separated by 8–10% sodium dodecyl sulfate–polyacrylamide gel electrophoresis (SDS-PAGE). Then, protein spots were transferred onto 0.2 μm polyvinylidene difluoride (PVDF) membrane (Bio-Rad Laboratories, CA, USA; Cat. No. 162-0177). The PVDF membrane was blocked with 5% bovine serum albumin (BSA) in Tris-buffered saline containing 0.1% Tween-20 (TBST) for 1 h at room temperature and then was incubated overnight at 4 °C with the primary antibodies: anti-VAV2 (1:1000; Proteintech, Cat. No. 21924-1-AP) and anti-GAPDH (1:5000; Abcam, Cat. No. ab8245). After three 10-minute washes with TBST, the membrane was incubated with horseradish peroxidase (HRP)-conjugated goat anti-rabbit IgG secondary antibody (1:10,000; Abcam, Cat. No. ab6721) for 1 h at room temperature. Protein bands were visualized using enhanced chemiluminescence (ECL) reagent and were imaged with a gel documentation system. Band intensities were quantified by densitometry using ImageJ software (version 1.52v, NIH, USA). VAV2 protein expression levels were normalized to GAPDH.

### Histopathological examination

Tissue specimens of LSCC, adjacent normal tissues, and neck dissection samples were fixed in 10% neutral-buffered formalin for 24–48 h and subsequently embedded in paraffin blocks. Sections of 4–5 μm thickness were prepared using a microtome and mounted on glass slides. The slides were deparaffinized in xylene and rehydrated through a graded ethanol series (absolute, 96%, 90%, and 80%). Nuclei were stained with hematoxylin for 5 min and differentiated in acid alcohol for 30 s, followed by rinsing with tap and distilled water. Counterstaining with eosin was performed for 30 s. The slides were then dehydrated in graded ethanol (80%, 90%, 96%, and absolute) and cleared in xylene, and then mounted. Finally, the stained sections were examined under a light microscope for histopathological examination [37].

### Statistics

All statistical analyses were conducted using IBM SPSS Statistics (version 26.0; IBM Corp., Armonk, NY, USA), GraphPad Prism (version 9.0; GraphPad Software, San Diego, CA, USA), and the R statistical environment (version 4.3.3; R Foundation for Statistical Computing, Vienna, Austria). The Kolmogorov–Smirnov test was used to assess data distribution, and nonparametric clinicopathological variables were compared using the Mann–Whitney U test. The Student’s t‑test was applied to paired parametric data. Correlations between gene, miRNA, and lncRNA expression levels were examined using Spearman’s rank correlation coefficient. Statistical significance was defined as *p* < 0.05. Pathway enrichment analysis was performed using the Benjamini–Hochberg false discovery rate adjustment and the Bonferroni correction to control for multiple testing. Kaplan–Meier survival analysis was performed on TCGA-HNSC patients (stages III–IV), using the optimal cut‑off of VAV2 expression to stratify cases into high‑ and low‑expression groups.

## Results

### Pathway enrichment analysis

**Fig. 1:**
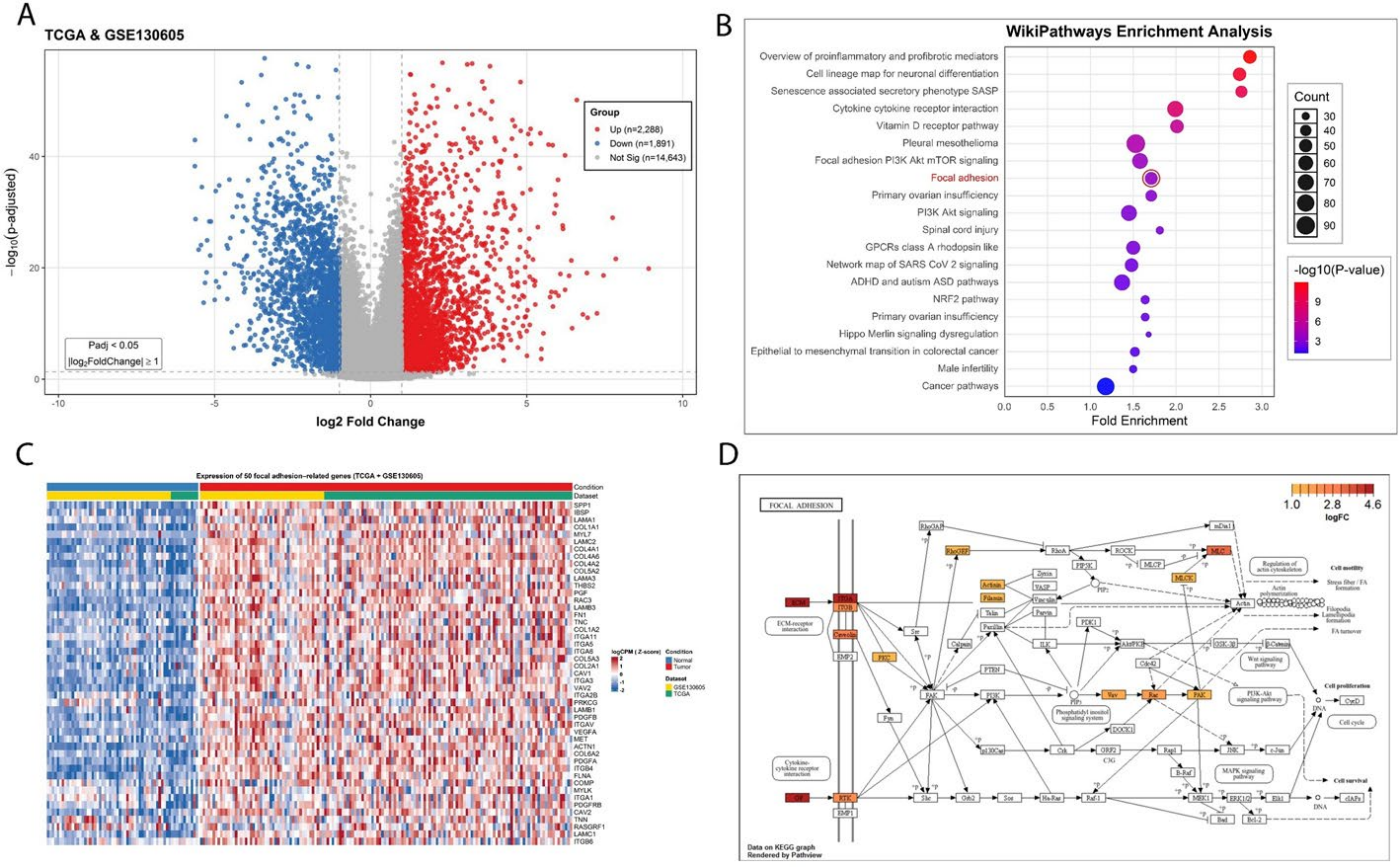
Gene and pathway enrichment analyses. **A** Volcano plot. **B** Enrichment plot. **C** Heatmap plot. **D** KEGG pathway (hsa04510)

After integrating the RNA-seq datasets from TCGA and GSE130605, among coding genes (*n* = 18,822), 2,288 genes were up-regulated (Fig. 1A). The pathway enrichment analysis identified the biological roles of the upregulated genes. The high-count signaling pathways (*n* = 20) ranked with P-value (Fig. 1B) included several pathways such as focal adhesion (*P* = 2.23 × 10⁻⁴, FDR = 1.16 × 10⁻², Fold Enrichment = 1.71) (Table 2). A total of 47 upregulated genes involved in the focal adhesion pathway were shown in a heatmap (Fig. 1C), mapped on the KEGG focal adhesion pathway (hsa04510), and color-scaled by log₂ fold-changes, through which VAV2 emerged as a centrally positioned and upregulated gene (Fig. 1D).

**Table 2:** Top Ten Pathways obtained from WIKIPATHWAYS Enrichment Analysis

Pathway		Count	*P*-Value	Benjamini	Fold Enrichment	Bonferroni	FDR
Overview of proinflammatory and profibrotic mediators		51	1.14E-12	8.73E-10	2.86	8.73E-10	8.32E-10
Cell lineage map for neuronal differentiation		50	1.24E-11	4.76E-09	2.74	9.52E-09	4.54E-09
Senescence associated secretory phenotype SASP		43	3.51E-10	8.96E-08	2.76	2.69E-07	8.54E-08
Cytokine cytokine receptor interaction		70	1.11E-08	2.12E-06	1.99	8.47E-06	2.02E-06
Vitamin D receptor pathway		52	7.13E-07	0.000091	2.01	0.000546	8.67E-05
Pleural mesothelioma		93	1.54E-05	0.00118	1.53	0.0117	0.00113
Focal adhesion PI3K Akt mTOR signaling		66	0.000137	0.00807	1.58	0.0996	0.00769
Focal adhesion		47	0.000223	0.0122	1.71	0.157	0.0116
Primary ovarian insufficiency		41	0.000628	0.024	1.71	0.382	0.0229
PI3K Akt signaling		68	0.0012	0.0399	1.45	0.601	0.038
Spinal cord injury		30	0.00149	0.0439	1.81	0.681	0.0418
GPCRs class A rhodopsin like		54	0.00188	0.0496	1.50	0.763	0.0473
Network map of SARS CoV 2 signaling		52	0.00315	0.0709	1.48	0.911	0.0676
ADHD and autism ASD pathways		70	0.00485	0.0929	1.37	0.976	0.0885
NRF2 pathway		32	0.005	0.0934	1.64	0.978	0.089
Primary ovarian insufficiency		31	0.00605	0.11	1.64	0.99	0.105
Hippo Merlin signaling dysregulation		28	0.00688	0.122	1.68	0.995	0.117
Epithelial to mesenchymal transition in colorectal cancer		34	0.0126	0.205	1.52	1.00e + 0	0.196
Male infertility		30	0.0234	0.311	1.50	1.00e + 0	0.296
Cancer pathways		83	0.0695	0.649	1.18	1.00e + 0	0.619

### Kaplan–Meier survival analysis

**Fig. 2:**
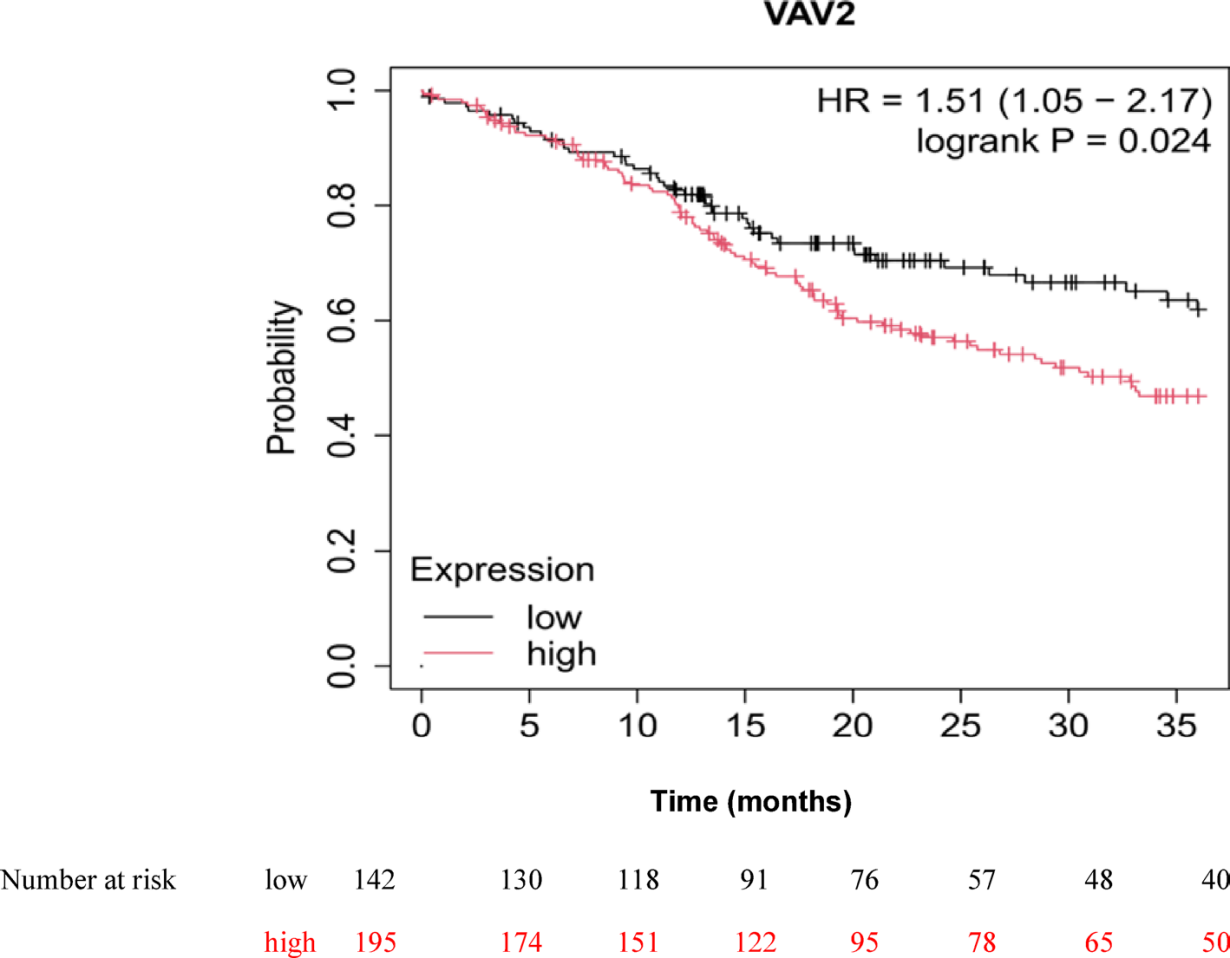
Kaplan–Meier survival analysis. Survival probability was evaluated in TCGA‑HNSC patientsstratified by VAV2 expression levels. HR, Hazard Ratio. Low (Black) and High (Red)

A Kaplan–Meier survival analysis showed that higher VAV2 expression levels were significantly associated with reduced survival probability over a 3‑year follow‑up period. Consistently, the estimated hazard ratio (HR) for VAV2 was 1.51 (95% CI: 1.05–2.17), indicating that patients with elevated VAV2 expression had approximately a 51% higher risk of adverse events compared to those with lower expression (Fig. 2).

### Gene ontology (GO) analysis of VAV2

**Fig. 3:**
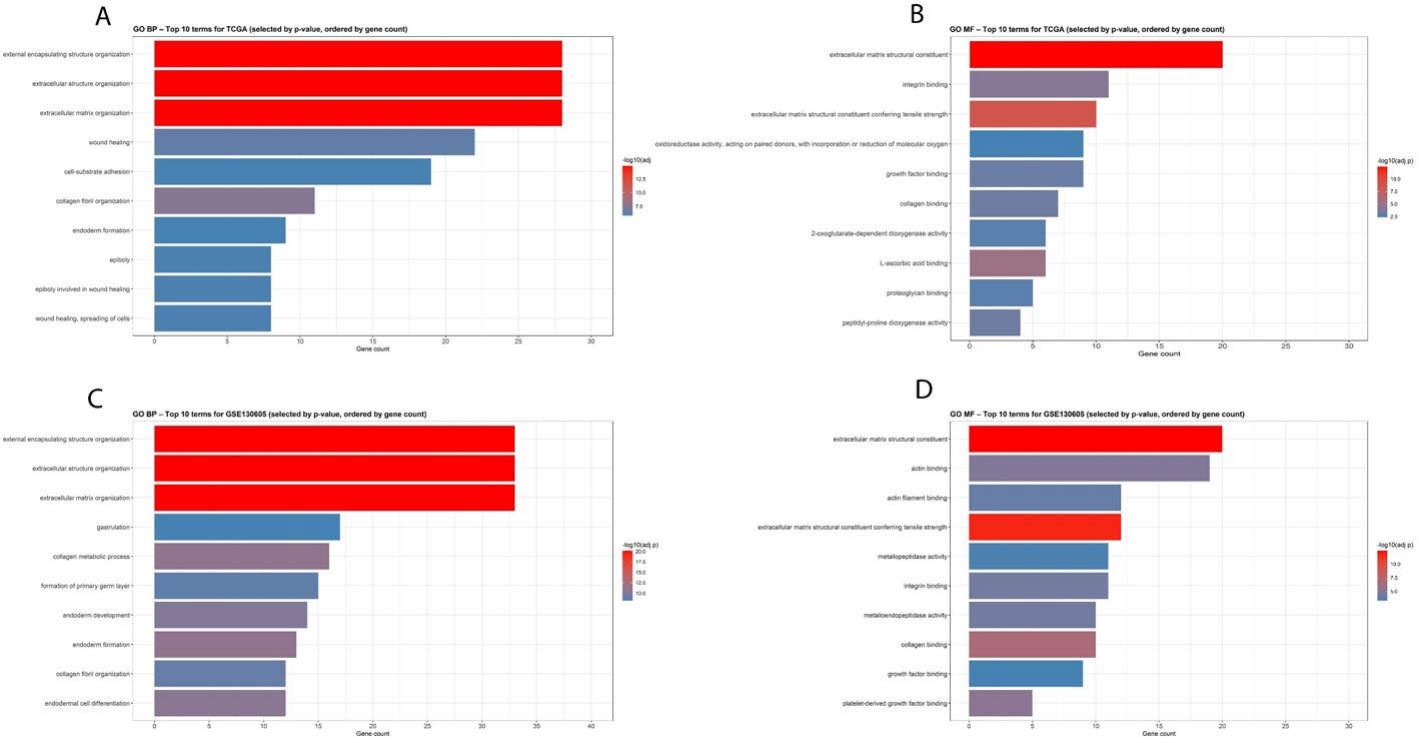
GO enrichment analysis of VAV2-coexpressed genes. **A** Top 10 GO Biological Process (BP) terms in the TCGA dataset. **B** Top 10 GO Molecular Function (MF) terms in the TCGA dataset. **C** Top 10 GO Biological Process (BP) terms in the GSE130605 dataset. **D** Top 10 GO Molecular Function (MF) terms in the GSE130605 dataset

Gene Ontology enrichment analysis was used to explore the biological functions associated with VAV2 in LSCC. For this purpose, the top 200 genes most strongly co-expressed with VAV2 were selected from the TCGA and GSE130605 datasets and analyzed using the clusterProfiler package in R to assess significantly overrepresented biological process and molecular function terms. The biological process results were mainly related to extracellular matrix organization, cell adhesion, and wound healing, indicating a role for VAV2 in tissue remodeling. Furthermore, molecular function analysis associated with extracellular matrix structural constituents, actin binding, and integrin binding suggested that VAV2 is involved in cell adhesion, cytoskeletal organization, and integrin-mediated signaling (Fig. 3).

###  VAV2/miRNA/lncRNA network

**Fig. 4:**
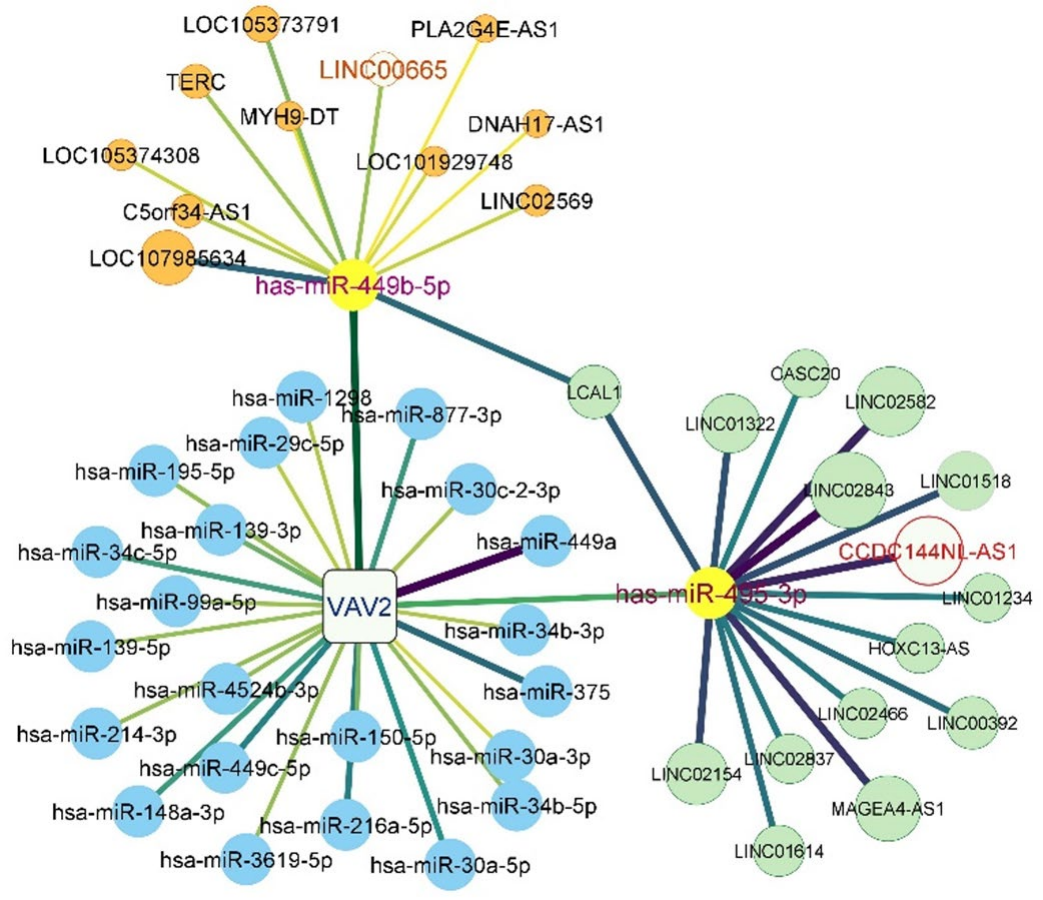
VAV2/miRNA/lncRNA network. The network edges and lncRNA node sizes are considered as the predicted scores. Edge: The lowest score (yellow) and the highest score (dark)

Based on database analysis, high‑score miRNAs were predicted to associate with VAV2. Among the top ten candidates, miR-449b-5p (|score|: 6.23) and miR-495-3p (|score|: 4.72) were selected, and their scores were visualized as network edges (Supplement 1, Fig. 3). In addition, high‑score lncRNAs were independently predicted for each of the miRNAs. CCDC144NL‑AS1 and LINC00665 were prioritized based on their high scores (5.568 and 3.959, respectively) and were incorporated into the network as edge and node size (Supplement 2 and 3, Fig. 4).

### VAV2, miRNA, and lncRNA gene expression levels in normal and LSCC tissues

**Fig. 5:**
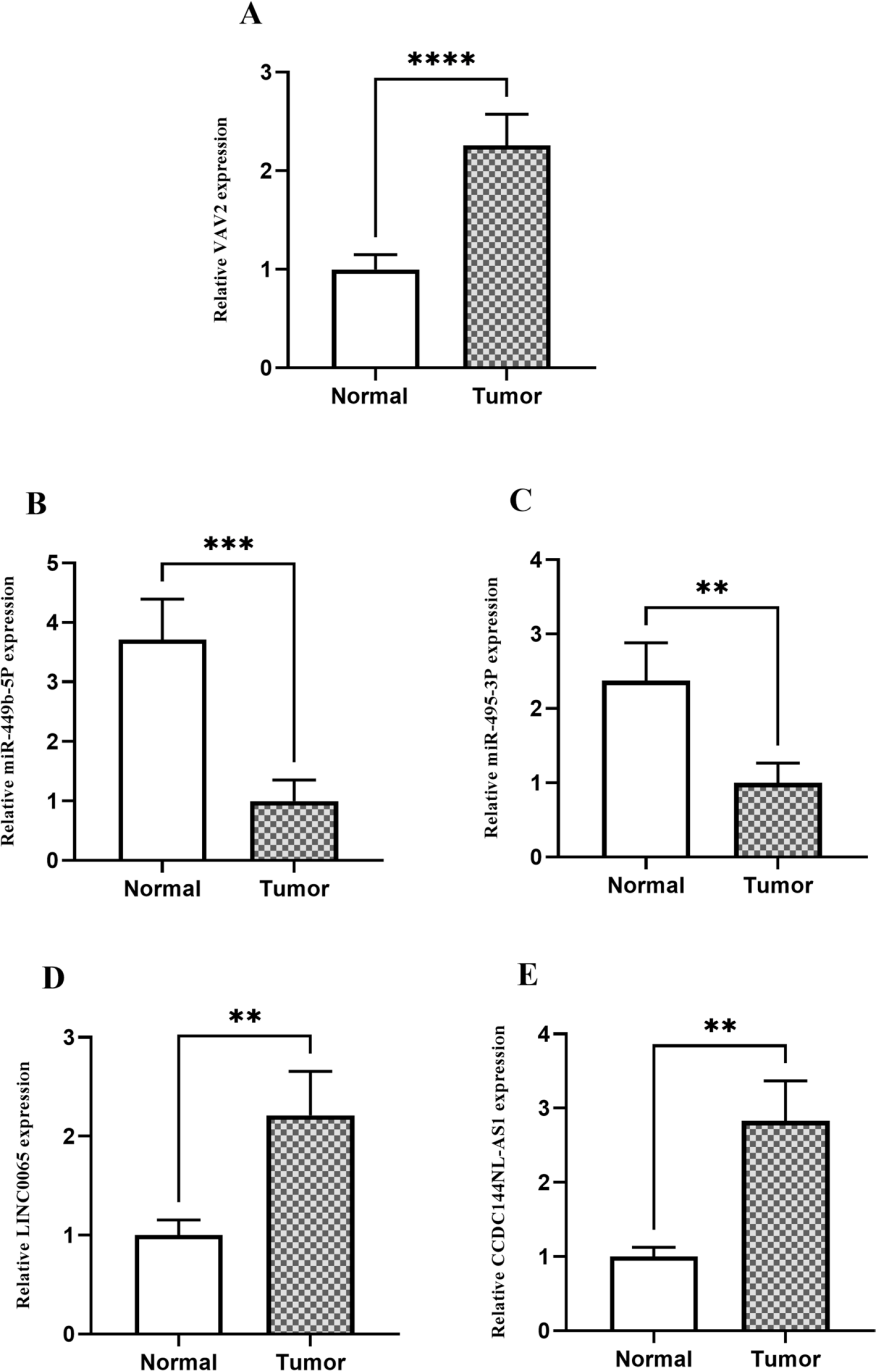
VAV2, miRNA, and lncRNA gene expression levels in normal and tumor tissues. **A** VAV2 (****P < 0.0001). **B** miR-449b-5p (***P < 0.001). **C** miR-495-3p (**P < 0.01). **D** LINC00665 (**P < 0.01). **E** CCDC144NL-AS1 (**P < 0.01). Data are presented as mean ± SEM

VAV2 gene expression levels were significantly higher in tumor tissues compared with matched normal tissues (*P* < 0.0001; Fig. 5A). Conversely, both miR-449b-5p and miR-495-3p were significantly reduced in tumor tissues (*P* < 0.001 and *P* < 0.01, respectively; Fig. 5B and C). Moreover, lncRNAs LINC00665 and CCDC144NL-AS1 showed significant upregulation in tumor samples (*P* < 0.01; Fig. 5D and E).

### VAV2 protein expression levels in normal and tumor tissues

**Fig. 6:**
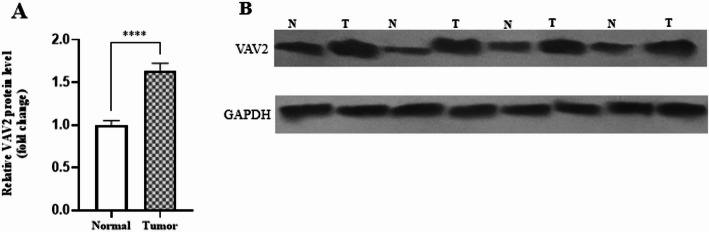
VAV2 protein expression levels in LSCC and adjacent normal tissues. **A** VAV2 protein expression levels (**** *P* < 0.0001). **B** Western blot image. Data are presented as mean ± SEM. N, Normal. T, Tumor

The VAV2 protein levels were markedly upregulated in tumor tissues compared to adjacent normal tissues (****P < 0.0001) (Fig. 6, Supplement 4). These findings showed that VAV2 protein expression is upregulated, in line with its gene expression levels in LSCC.

### Tumor characteristics

**Fig. 7:**
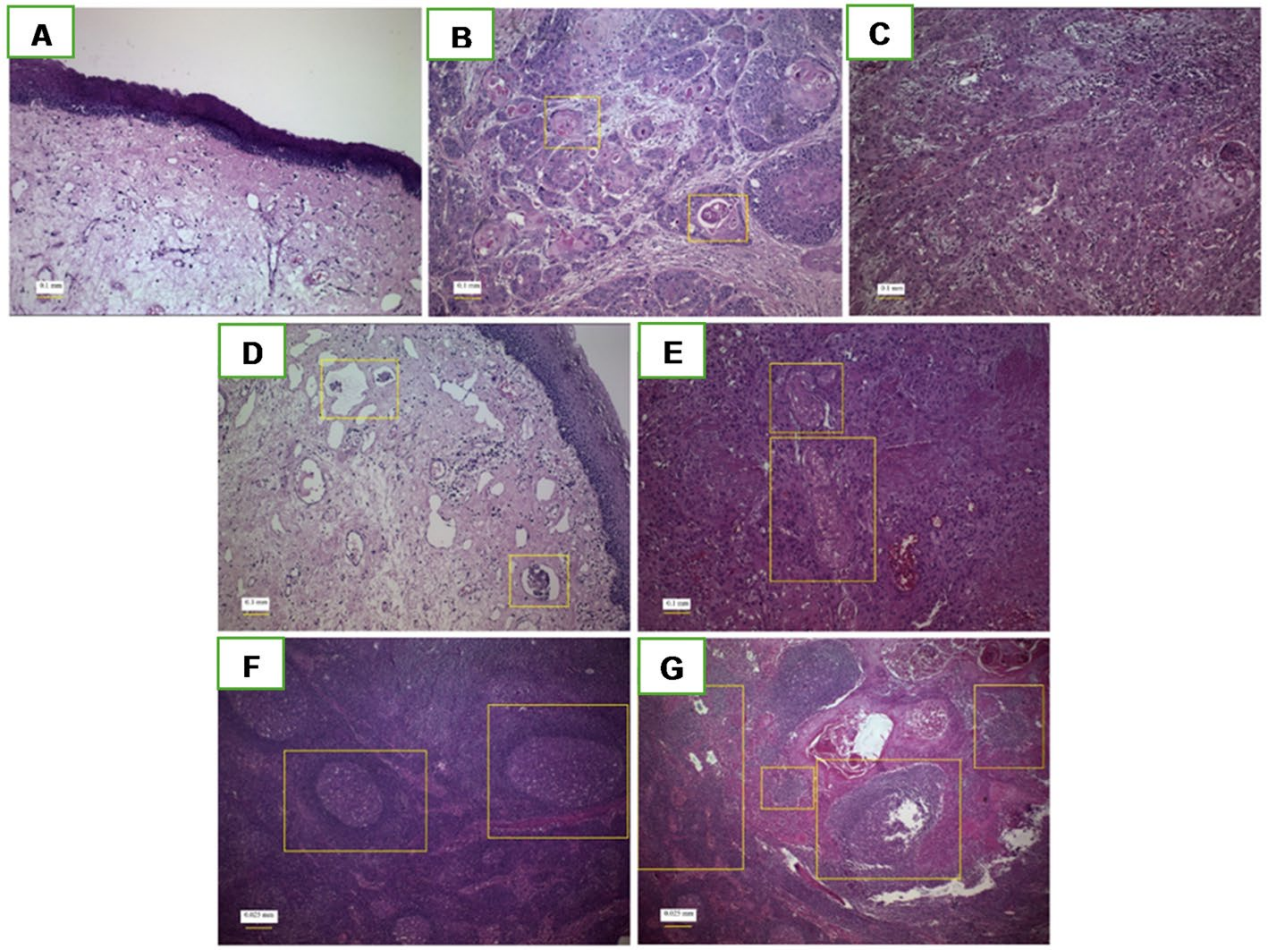
Histopathological features of LSCC tissues. **A** Normal laryngeal squamous epithelial cells, without dysplasia or neoplastic infiltration (Scale bar: 0.1 mm). **B** Laryngeal tissue involved by well-differentiated SCC with keratin pearl formation and organized tumor nests. The yellow rectangles show keratin pearls (Scale bar: 0.1 mm). **C** Laryngeal tissue with moderately differentiated SCC, showing disrupted maturation and irregular invasive tumor nests (scale bar: 0.1 mm). **D** Tumor cell infiltration of lymphatic and vascular channels (lymphovascular invasion) (scale bar: 0.1 mm). **E** Tumor cells encasing and invading nerve fibers (perineural invasion) (scale bar: 0.1 mm). **F** Normal lymph node with preserved follicular architecture and absence of tumor cells. The yellow rectangles show germinal centers (Scale bar: 0.025 mm). **G** Lymph node involvement, demonstrating metastatic LSCC cells replacing normal nodal architecture (Scale bar: 0.025 mm)

The study consisted of 63 male patients with an average age of 57.9 ± 8.85 (mean ± SD) years. Histopathological evaluations compared with normal tissues (Fig. 7A) estimated that 41 tumors (65.08%) were well differentiated (Fig. 7B), whereas 22 tumors (34.92%) were moderately differentiated (Fig. 7C). Vocal cord invasion was observed in 41 patients (65.08%), anterior commissure involvement in 32 patients (50.79%), epiglottic invasion in 38 patients (60.32%), and thyroid cartilage invasion in 35 patients (55.56%). Moreover, extralaryngeal soft-tissue extension was present in 23 patients (36.51%). Additional adverse pathological features included lymphovascular invasion in 15 cases (23.81%) (Fig. 7D) and perineural infiltration in 20 cases (31.75%) (Fig. 7E). According to clinical staging, 28 patients (44.44%) were classified as stage III and 35 patients (55.56%) as stage IV. Regional lymph node metastasis was absent in 46 cases (73.1%; Fig. 7F), whereas 17 patients (26.9%) showed nodal involvement (Fig. 7G), with details provided in Table 3.

### Associations between clinicopathological parameters and gene expression levels

The VAV2, miR-495-3p, miR-449b-5p, LINC00665, and CCDC144NL-AS1 gene expression levels were evaluated between subgroups (Table 3). No significant associations were detected with age, T stage, histological differentiation, lymph node involvement, and patterns of local invasion, including the thyroid cartilage, anterior commissure, epiglottis, and vocal cords (*P* > 0.05). By contrast, several lifestyle-related variables showed significant relationships. Smoking was related to lower expression of miR-495-3p (*P* = 0.0021) and miR-449b-5p (*P* = 0.0006), and alcohol consumption was related to reduced miR-449b-5p expression (Median 0.03 (Yes)/1.08 (No), *P* = 0.0036). Opium use was linked to decreased expression of both VAV2 (Median 1.06 (Yes)/1.6 (No), *P* = 0.0398) and CCDC144NL-AS1 (Median 0.5 (Yes)/1.87 (No), *P* = 0.0010). In addition, higher CCDC144NL-AS1 expression was significantly associated with extralaryngeal soft tissue invasion (*P* = 0.0428).

**Table 3:** Associations Between Clinicopathological Parameters and Gene Expression Levels

Characteristic	Category	Frequency (*n*, %)	VAV2 (*p* value*)	miR-495-3p (*p* value*)	miR-449b-5p (*p* value*)	LINC00665 (*p* value*)	CCDC144NL-AS1 (*p* value*)
Age (Year)	≤ 60 > 60	43 (68.3%) 20 (31.7%)	0.3364	0.6657	0.6657	0.0882	0.1096
T stage	III IV	28 (44.4%) 35 (55.6%)	0.7056	0.6752	0.5685	0.6355	0.3117
Lymph node metastasis	Yes No	17 (26.9%) 46(73.1%)	0.4656	0.8360	0.7884	0.7766	0.8240
Differentiation of cancer tissue	Well Moderate	41 (65.1%) 22 (34.9%)	0.2600	0.5335	0.6730	0.4435	0.7260
Thyroid cartilage involvement	Yes No	35 (55.6%) 28(44.4%)	0.7056	0.6752	0.5685	0.6355	0.3117
Anterior commissure involvement	Yes No	32 (50.8%) 31 (49.2%)	0.5895	0.8006	0.7175	0.8431	0.6181
Extra laryngeal soft tissues invasion	Yes No	23 (36.5%) 40 (63.5%)	0.2586	0.9492	0.9042	0.3985	0.0428
Lymphovascular invasion	Yes No	15 (23.8%) 48 (76.2%)	0.8169	0.7434	0.6838	0.3830	0.6148
Perineural invasion	Yes No	20 (31.7%) 43 (68.3%)	0.3218	0.1981	0.3748	0.6236	0.5334
Epiglottis invasion	Yes No	38 (60.3%) 25(39.7%)	0.3456	0.6503	0.1591	0.3981	0.2316
Vocal cord involvement	Yes No	41 (65.1%) 22(34.9%)	0.1654	0.6215	0.1654	0.1183	0.1183
Smoking	Yes No	51 (80.9%) 12 (19.1%)	0.7623	0.0021	0.0006	0.9518	0.8156
Alcohol consumption	Yes No	5 (7.9%) 58 (92.1%)	0.4958	0.0602	0.0036	0.1630	0.1879
Opium consumption	Yes No	23 (36.5%) 40 (63.5%)	0.0398	0.7607	0.0994	0.0936	0.0010

*Mann–Whitney U test

### Gene correlations

**Fig. 8:**
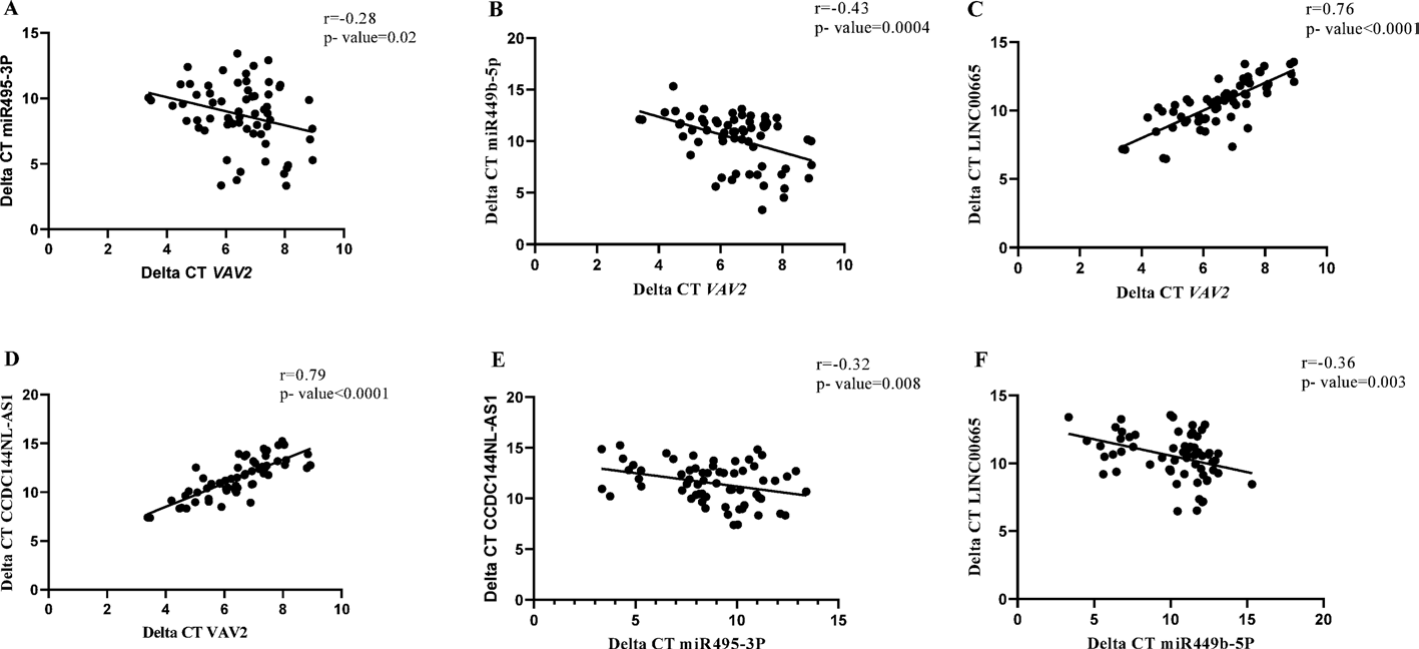
VAV2 and ncRNAs correlations. **A** VAV2 vs. miR-495-3p. **B** VAV2 vs. miR-449b-5p. **C** VAV2 vs. LINC00665. **D** VAV2 vs. CCDC144NL-AS1. **E** CCDC144NL-AS1 vs. miR-495-3p. **F** LINC00665 vs. miR-449b-5p

VAV2 expression levels showed significant negative correlations with miR-495-3p (*r* = − 0.28, *P* = 0.02) and miR-449b-5p (*r* = − 0.43, *P* = 0.0004) and significant positive correlations with LINC00665 (*r* = 0.76, *P* < 0.0001) and CCDC144NL-AS1 (*r* = 0.79, *P* < 0.0001) (Fig. 8). LINC00665 and miR-449b-5p (*r* = − 0.36, *P* = 0.003) as well as CCDC144NL-AS1 and miR-495-3p (*r* = − 0.32, *P* = 0.008) exhibited the inverse relationships.

## Discussion

The bioinformatics data suggested focal adhesion signaling as one of the dysregulated pathways in LSCC. This pathway may serve for invasion and metastasis in squamous carcinomas, as it modulates extracellular matrix remodeling, cytoskeletal dynamics, and integrin-mediated motility. VAV2 has been recognized as a central gene in this pathway, which is proposed to link integrin contacts to actin cytoskeletal remodeling, acting in tumor cell motility.

Gene Ontology analyses highlighted the functional importance of VAV2 in processes such as extracellular matrix organization, cell adhesion, actin binding, and integrin binding, which are essential contributors to tumor progression. Moreover, higher VAV2 expression levels significantly reduced survival probability. In agreement with our results, VAV2 overexpression is reported in the development of ductal carcinoma in situ to invasive breast cancer [17], enhanced gastric cancer invasion and metastasis [19], and promoted radiation resistance through DNA repair [38]. It also regulated cell adhesion via VAV2–Rac1–FAK signaling in lung cancer [20], mediated cell proliferation through the VAV2/Rac1 axis in breast carcinoma [39], interacted with AFAP1L1 to activate ITGA5 signaling in gastric cancer [40], induced regenerative proliferation and stem-like programs in head and neck squamous cell carcinoma [23, 24], and stabilized AR/ARv7 to confer enzalutamide. A comprehensive study showed its overwhelming carcinogenic significance in cytoskeletal remodeling and focal adhesion turnover, with few tumor-suppressive effects [41].

In the weighted scoring approach, miR-449b-5p and miR-495-3p were predicted to associate with the VAV2. Furthermore, both miR-449b-5p and miR-495-3p were found to be downregulated in LSCC tissues and were widely acknowledged as tumor suppressors in various malignancies [42–45]. miR-449b-5p influenced pathways related to proliferation, migration, and cytoskeletal organization [46–48], while miR-495-3p regulated oncogenic processes that lead to growth, invasion, therapeutic resistance, and epithelial–mesenchymal plasticity [49–51]. Considering this miRNA regulatory profile, it is reasonable to infer that lncRNA regulators may also play a role in the dysregulation of this axis in LSCC [34], hence justifying the investigation of lncRNA–miRNA interactions within the extensive VAV2 regulatory network.

Both LINC00665 and CCDC144NL-AS1 were significantly upregulated in LSCC tissues compared to adjacent normal tissues. LINC00665 has been reported in various malignancies as an oncogenic lncRNA that predominantly functions by sponging a wide array of tumor-suppressive miRNAs. By sequestering miRNAs, LINC00665 allowed the number of oncogenic transcripts to escape repression, which in turn led to the activation of several cancer-related signaling pathways, including Wnt/β-catenin, TGF-β/Smad, PI3K/AKT, NF-κB, and MAPK/ERK. These signaling events were associated with increased cell proliferation and invasion, as well as EMT, altered glycolytic activity, and resistance to chemotherapy [52–55]. CCDC144NL-AS1 is reported to play a comparable oncogenic role. It is directly bonded to miR-143-3p, resulting in elevated MAP3K7 expression [56], interacts with miR-145-5p in the context of SERPINE1 regulation, and sponges with miR-490-3p to promote HMGA2 expression [57, 58]. These combined interactions caused the aberrant activation of several oncogenic pathways, particularly MAPK/ERK, PI3K/AKT, and Wnt/β-catenin [56, 59].

The co‑overexpression of LINC00665 and CCDC144NL‑AS1 in LSCC, associated with their inverse correlation with miR‑449b‑5p and miR‑495‑3p, suggests their contribution to tumor progression and their potential utility in diagnosis, prognosis, and targeted therapy [60, 61]. The study showed that smoking has a significant association with lower expression of the miR-495-3p and miR-449b-5p. Moreover, the findings showed that the CCDC144NL-AS1and VAV2 expression levels were significantly different among patients consuming opium. While some opium alkaloids have been reported to exert antiproliferative effects in vitro models, opium use is clearly associated with increased cancer risk in epidemiologic studies. Furthermore, the results were limited by subgroup sizes, indicating that validation should be obtained in larger and more balanced cohorts. Therefore, the observed association between alcohol, opium, and expression levels of ncRNAs and VAV2 may reflect residual confounding rather than a biological effect [62]. Higher levels of CCDC144NL-AS1 expression were strongly linked to invasion into soft tissues outside of the larynx, so that it may play a role in the invasive and locoregionally aggressive character of LSCC.

## Conclusion

Our approach integrated both TCGA and GEO datasets, applying stricter differential‑expression and interaction‑filtering criteria, and incorporated multiple predictive and experimentally validated interaction databases to strengthen the reliability of the gene/miRNA/lncRNA network. In addition, we included clinical validation to enhance the biological and translational relevance of the bioinformatic findings. The study suggested that the focal adhesion signaling pathway is dysregulated in LSCC, likely through central genes such as VAV2. By integrating bioinformatics databases with experimental data, the results suggested previously unknown axes regulating VAV2 by LINC00665/miR-449b-5p and CCDC144NL-AS1/miR-495-3p in tumor tissues. The co-expression of VAV2 and lncRNAs, together with reduced expression of miRNAs in the LSCC population, proposed a model in which LINC00665 and CCDC144NL-AS1 may act as molecular sponges for miR-449b-5p and miR-495-3p. Although the data revealed novel molecular insights into ncRNAs and VAV2, the limited sample size highlights the need for validation in a larger, comprehensive cohort. Moreover, to further validate the proposed ceRNA model, targeted assays—including luciferase reporter, RNA pull‑down, miRNA mimic/inhibitor transfection, and lncRNA expression control with appropriate experiments—should confirm whether LINC00665/miR‑449b‑5p and CCDC144NL‑AS1/miR‑495‑3p directly regulate VAV2 and influence cellular behavior.

## Supplementary Information

Supplementary Material 1

Supplementary Material 2

Supplementary Material 3

Supplementary Material 4

## Data Availability

The RNAseq data are obtained from TCGA and GEO databases and are freely available at (https:/www.cancer.gov/ccg/research/genome-sequencing/tcga) and (https:/www.ncbi.nlm.nih.gov/geo). The pathway enrichment is freely available at https://davidbioinformatics.nih.gov. The ncRNA databases are freely available at miRTarBase (https:/mirtarbase.cuhk.edu.cn), StarBase (https:/rnasysu.com/encori), DIANA-TarBase (https:/dianalab.e-ce.uth.gr/home), TargetScan (https:/www.targetscan.org), miRWalk (http:/mirwalk.umm.uni-heidelberg.de), miRDB (https:/mirdb.org), and miRmap (https:/mirmap.ezlab.org) and LncBase v3.0 (https:/diana.e-ce.uth.gr/lncbasev3).

## Abbreviations

AFAP1L1: Actin filament associated protein 1 like 1
AKR1B10: Aldo-keto reductase family 1 member B10
AKT: Protein kinase B
AR: Androgen receptor
ARv7: Androgen receptor variant 7
ATF1: Activating transcription factor 1
BCL9L: BCL9 like
BP: Biological process
BSA: Bovine serum albumin
CCDC144NL-AS1: Coiled-coil domain containing 144 N-terminal like antisense RNA 1
ceRNA: Competing endogenous RNA
cDNA: Complementary DNA
CIP2A-BP: Cancer inhibitor of PP2A - binding protein
COL1A1: Collagen type I Alpha 1 chain
Ct: Cycle threshold
CTNNB1: Catenin beta 1 (β-catenin)
Cytoscape: Cytoscape software
DAVID: Database for annotation, visualization and integrated discovery
DEG: Differentially expressed Gene
DElncRNA: Differentially expressed lncRNA
DEmiR: Differentially expressed miRNA
DIANA-TarBase: DIANA-TarBase database
ECL: Enhanced chemiluminescence
EMT: Epithelial-mesenchymal transition
ERK: Extracellular signal-regulated kinase
FAK: Focal adhesion kinase
FDR: False discovery rate
FN: Fibronectin 1
GAPDH: Glyceraldehyde-3-phosphate dehydrogenase
GC: Gastric cancer
GEO: Gene expression omnibus
GO: Gene ontology
HCC: Hepatocellular carcinoma
HMGA2: High mobility group AT-hook 2
HNSCC: Head and neck squamous cell carcinoma
HRP: Horseradish peroxidase
hsa04510: KEGG pathway ID for human focal adhesion
IgG: Immunoglobulin G
ITGA5: Integrin subunit alpha 5
KEGG: Kyoto encyclopedia of genes and genomes
LAMC2: Laminin subunit gamma 2
LncBase: LncBase database
LINC00665: Long intergenic non-protein coding RNA 665
lncRNA: Long non-coding RNA
logCPM: log2 counts per million
LSCC: Laryngeal squamous cell carcinoma
MAP3K7: Mitogen-activated protein kinase kinase kinase 7
MAPK: Mitogen-activated protein kinase
MF: Molecular function
miRNA: microRNA
miRDB: miRDB database
miRmap: miRmap database
miRTarBase: miRTarBase database
miRWalk: miRWalk database
NCBI: National Center for Biotechnology Information
NF-κB: Nuclear Factor kappa B
NIH: National Institutes of Health
NSCLC: Non-small cell lung cancer
OC: Ovarian cancer
PI3K: Phosphoinositide 3-Kinase
PP2A: Protein phosphatase 2 A
PVDF: Polyvinylidene difluoride
qRT-PCR: Quantitative real-time polymerase chain reaction
Rac1: Ras-Related C3 botulinum toxin substrate 1
RIPA: Radioimmunoprecipitation assay buffer
SCC: Squamous cell carcinoma
SD: Standard deviation
SDS-PAGE: Sodium dodecyl sulfate–polyacrylamide gel electrophoresis
SEM: Standard error of the mean
SERPINE1: Serpin family E member 1
SPP1: Secreted Phosphoprotein 1 (Osteopontin)
SPSS: Statistical package for the social sciences
StarBase: StarBase database
STAD: Stomach adenocarcinoma
SYBR: SYBR green
TargetScan: TargetScan database
TBST: Tris-buffered saline with tween-20
TCGA: The cancer genome atlas
TGF-β: Transforming growth factor beta
TMM: Trimmed mean of M-values
TNC: Tenascin C
U2AF2: U2 small nuclear RNA auxiliary factor 2
U6: U6 small nuclear RNA
VEGFA: Vascular endothelial growth factor A
VAV2: Vav guanine nucleotide exchange factor 2
WDR5: WD repeat domain 5
