# Supplementary material for: VAV2-associated ncRNA network in the focal adhesion pathway is dysregulated in laryngeal squamous cell carcinoma
Source: Discov Oncol. 2026 May 27;17:1064. doi: 10.1007/s12672-026-05181-z (PMC13391986; doi:10.1007/s12672-026-05181-z)
Supplement: Supplementary file 4 — Supplementary Material 4 [file 12672_2026_5181_MOESM4_ESM.pptx]

## Slide 1
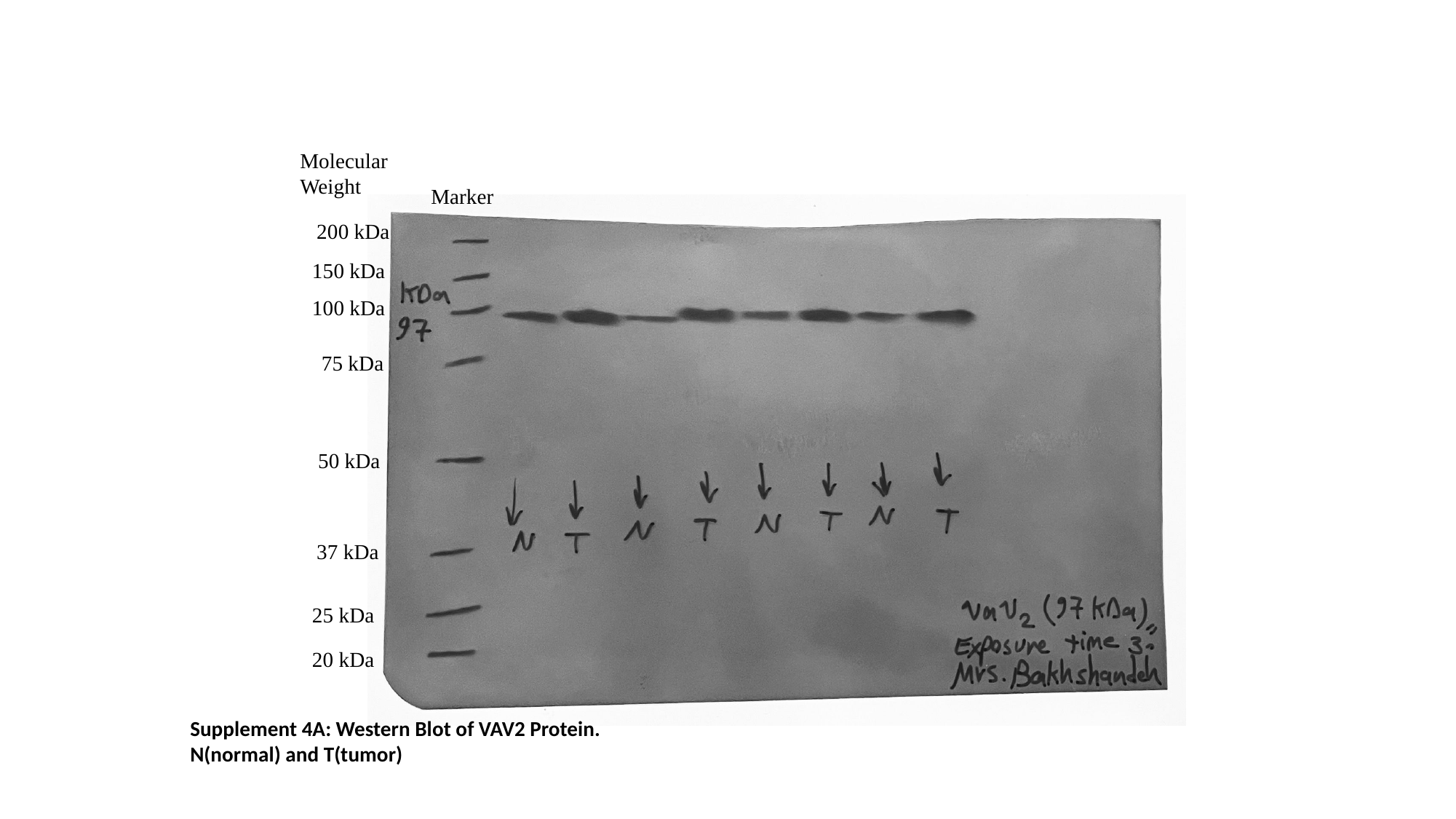

Molecular Weight
Marker
200 kDa
150 kDa
100 kDa
75 kDa
50 kDa
37 kDa
25 kDa
20 kDa
Supplement 4A: Western Blot of VAV2 Protein.
N(normal) and T(tumor)

## Slide 2
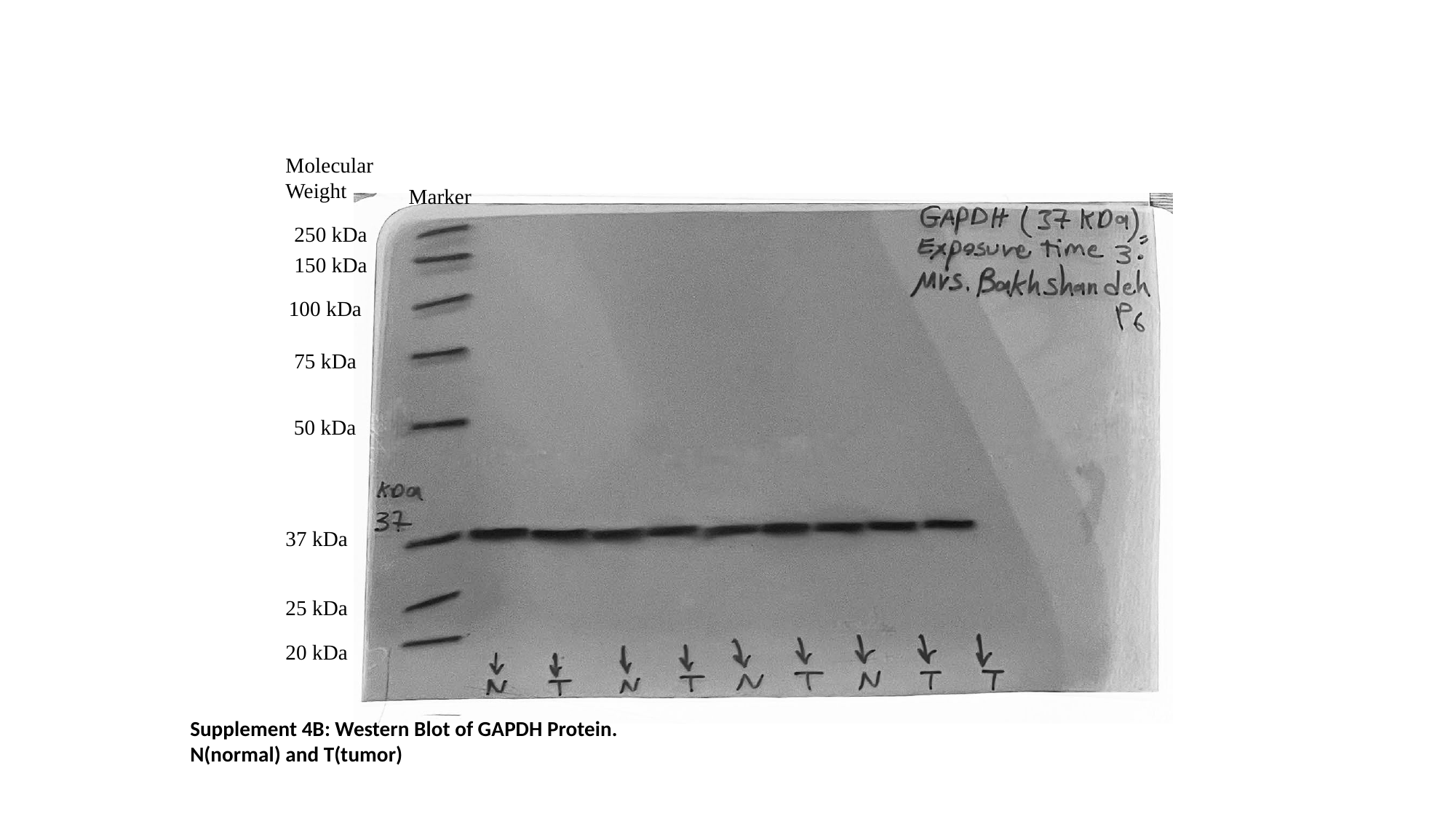

Molecular Weight
Marker
250 kDa
150 kDa
100 kDa
75 kDa
50 kDa
37 kDa
25 kDa
20 kDa
Supplement 4B: Western Blot of GAPDH Protein.
N(normal) and T(tumor)
